# Mitochondrial Strokes: Diagnostic Challenges and Chameleons

**DOI:** 10.3390/genes12101643

**Published:** 2021-10-19

**Authors:** Chiara Pizzamiglio, Enrico Bugiardini, William L. Macken, Cathy E. Woodward, Michael G. Hanna, Robert D. S. Pitceathly

**Affiliations:** 1 Department of Neuromuscular Diseases, UCL Queen Square Institute of Neurology and The National Hospital for Neurology and Neurosurgery, London WC1N 3BG, UK; c.pizzamiglio@ucl.ac.uk (C.P.); e.bugiardini@ucl.ac.uk (E.B.); w.macken@ucl.ac.uk (W.L.M.); m.hanna@ucl.ac.uk (M.G.H.); 2 Neurogenetics Unit, The National Hospital for Neurology and Neurosurgery, London WC1N 3BH, UK; cathywoodward@nhs.net

**Keywords:** primary mitochondrial diseases, MELAS, stroke-like episodes, brain MRI, mitochondrial DNA

## Abstract

Mitochondrial stroke-like episodes (SLEs) are a hallmark of mitochondrial encephalomyopathy, lactic acidosis, and stroke-like episodes (MELAS). They should be suspected in anyone with an acute/subacute onset of focal neurological symptoms at any age and are usually driven by seizures. Suggestive features of an underlying mitochondrial pathology include evolving MRI lesions, often originating within the posterior brain regions, the presence of multisystemic involvement, including diabetes, deafness, or cardiomyopathy, and a positive family history. The diagnosis of MELAS has important implications for those affected and their relatives, given it enables early initiation of appropriate treatment and genetic counselling. However, the diagnosis is frequently challenging, particularly during the acute phase of an event. We describe four cases of mitochondrial strokes to highlight the considerable overlap that exists with other neurological disorders, including viral and autoimmune encephalitis, ischemic stroke, and central nervous system (CNS) vasculitis, and discuss the clinical, laboratory, and imaging features that can help distinguish MELAS from these differential diagnoses.

## 1. Introduction

Mitochondrial stroke-like episodes (SLEs) are defined as acute/subacute, evolving, focal neurologic deficits, usually driven by seizure activity, in primary mitochondrial diseases (PMDs) [1], and are the hallmark of mitochondrial encephalomyopathy, lactic acidosis, and stroke-like episodes (MELAS). The mitochondrial DNA (mtDNA) mutation m.3243A > G in the *MT-TL1* gene was the first gene defect to be linked with MELAS [2]. However, many other pathogenic variants in mtDNA, e.g., m.3271T > C in *MT-TL1* and m.13513G > A in *MT-ND5* [3,4], and in nuclear genes, e.g., *POLG*, have since been reported in patients with mitochondrial strokes [5].

The m.3243A > G mutation is the most common cause of MELAS, with a prevalence of 10.6/100,000 [6]. According to the diagnostic criteria of MELAS [7], a combination of clinical findings of SLE plus evidence of mitochondrial dysfunction (i.e., definitive gene mutation related to MELAS, mitochondrial abnormalities in muscle biopsy, high lactate levels in plasma and/or in cerebral spinal fluid, or deficiency of mitochondrial enzyme activities) are necessary for a definite diagnosis. SLEs are usually characterised by headache, nausea and vomiting, altered conscious level or confusion, visual field defects, language impairment, focal motor weakness, or sensory symptoms. Epileptic seizures are often present and brain imaging may demonstrate stroke-like lesions that do not conform to a specific vascular territory [8]. SLEs are usually recurrent and lead to serious long-term consequences, including neurodegeneration, brain atrophy, and cognitive impairment [9].

The diagnosis can be extremely challenging during the acute phase of an SLE, particularly when the patient is presenting with MELAS for the first time. In addition to similarities with ischaemic strokes, MELAS may mimic autoimmune and infectious encephalopathies, including Hashimoto encephalopathy, anti-NMDA receptor encephalitis, and herpes simplex virus (HSV) encephalitis [10,11,12]. The accuracy of the diagnosis is also influenced by the experience of the evaluator and the timing of the assessment [13], thus indicating the importance of informing patients, caregivers, and clinicians about the nature of SLE once a diagnosis is confirmed.

Here, we describe four patients with MELAS presenting with an SLE that initially masqueraded as other acute/subacute acquired neurologic disorders, and highlight the importance of early diagnosis and appropriate management of SLEs to improve outcomes in MELAS.

## 2. Materials and Methods

All four patients are under active follow up in the NHS England Highly Specialised Service for Rare Mitochondrial Disorders, the National Hospital for Neurology and Neurosurgery (NHNN), London, UK.

Clinical evaluation, laboratory tests, MRI, and tissue biopsies were conducted as part of routine care, and investigations were analysed using standard protocols. Spectrophotometric analysis of mitochondrial respiratory chain enzyme activities (RCEA) as a ratio to citrate synthase activity was measured in skeletal muscle homogenate [14].

DNA was extracted from peripheral blood leukocytes using a manual salting-out method (Qiagen Flexigene kit, Qiagen, Düsseldorf, Germany) following the manufacturer’s instructions. DNA was extracted from urinary epithelial cell pellets and muscle tissue manually with the Promega Wizard^®^ Genomic DNA Purification Kit (Promega, Madison, WI, USA) using the ‘modified mouse tail protocol’.

Genetic analysis was performed either by sequencing of mtDNA or by fluorescent restriction fragment length polymorphism (RFLP) analysis for m.3243A > G. The entire mtDNA length was sequenced using the Illumina MiSeq [14]. Coverage of the mtDNA coding region was at a minimum read depth of 500×. Variant detection sensitivity was greater than 95% (95% confidence interval). Capillary electrophoresis of fluorescently labelled PCR products following restriction endonuclease digestion was used to confirm sequencing results and to quantify mutant load. The heteroplasmy level for both detection methods is ≥1%.

## 3. Results

Table 1 summarises the clinical, radiological, and molecular characteristics of the four subjects with SLEs caused by the m.3243A > G mutation in *MT-TL1*, outlined in further detail below.

### 3.1. Case 1

A 61-year-old woman of mixed Kuwaiti and Iraqi descent was hospitalised after a few days of progressive balance disturbance and drowsiness. Conscious level fluctuated (Glasgow Coma Scale (GCS) ranged from 3 to 13/15) and intermittent focal onset seizures were observed. Viral encephalitis was initially suspected based on the clinical presentation, with subacute onset of clouding of consciousness and seizures, although it was subsequently excluded based on normality of cerebrospinal fluid (CSF) constituents and negative viral polymerase chain reaction (PCR) for HSV 1 and 2, varicella zoster virus (VZV), cytomegalovirus, and enterovirus. Paraneoplastic antibodies were negative. CSF showed a moderate increase in lactate (4.5, reference range 1.1–2.4 mmol/L). She subsequently developed abdominal pseudo-obstruction requiring flatus tube insertion on two occasions.

**Figure 1:**
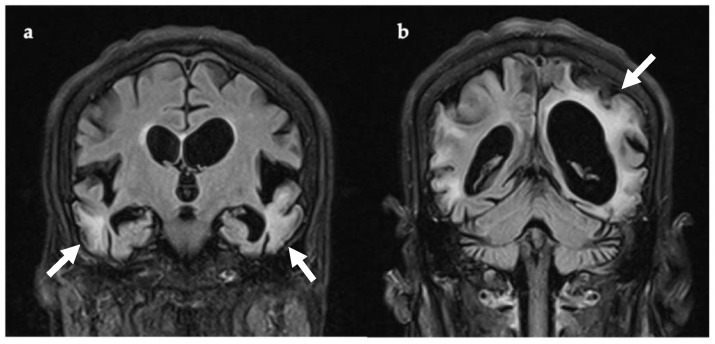
Coronal T2-FLAIR MRI, 21 days after symptom onset. There are multiple areas of supra-tentorial encephalomalacia bilaterally within the temporo-parieto-occipital lobes with parenchymal T2 signal change, thinning of gyri, and white matter volume loss ((**a**,**b**), white arrows). Multiple vascular territories are involved. Abbreviations: FLAIR, fluid attenuated inversion recovery imaging.

Following clinical deterioration, she was transferred to NHNN. Brain MRI (three weeks after symptom onset, Figure 1) showed multi-focal areas of recent parenchymal insult, characterised by cortical and subcortical white matter signal abnormalities and mild parenchymal swelling, affecting different vascular territories. The EEG had a slow background with no seizure activity, but occasional left temporal sharp waves.

Past medical history included two episodes of subacute neurological disturbance at the age of 51 and 56 years. During the first, she developed left-sided numbness and hemiplegia that progressed over seven days, followed by a focal to bilateral tonic-clonic seizure. The left-sided hemiplegia resolved after one week. In the second episode, she developed expressive dysphasia and drowsiness, worsening over one week. She was subsequently left with residual expressive dysphasia for English, Swedish, and native Arabic. At the age of 58 years she had a cluster of seizures, which were treated with anticonvulsants. She was diagnosed with diabetes at the age of 32 years and there was a history of hearing loss over the preceding year. An older brother and sister had a positive history of diabetes and stroke, respectively, at the age of 65 and 70 years. Her mother died aged 38 years during childbirth. She had one asymptomatic child.

Given the history of recurrent stroke-like episodes, diabetes, deafness, and MRI findings, MELAS was suspected. The m.3243A > G mutation was undetectable in blood, but was confirmed in urinary epithelial cells at a heteroplasmy level of 29%.

During her admission, she was initially hyperglycaemic (blood sugars 22, reference range 3.9–5.9 mmol/L), had a persistent lactic acidosis, and required insulin and intravenous hydration. Antiepileptic medications were optimised and she was discharged for rehabilitation. Unfortunately, she died at the age of 62 years following complication of MELAS. Confirmation of the diagnosis had direct implications for her daughter, who was confirmed to be a carrier of the m.3243A > G mutation (heteroplasmy unknown).

### 3.2. Case 2

A 51-year-old Vietnamese woman, resident in the UK for the past 20 years, presented to her local hospital with a 72-h history of confusion. Prior to this she was independent, with a normal cognitive profile and no previous similar episodes.

**Figure 2:**
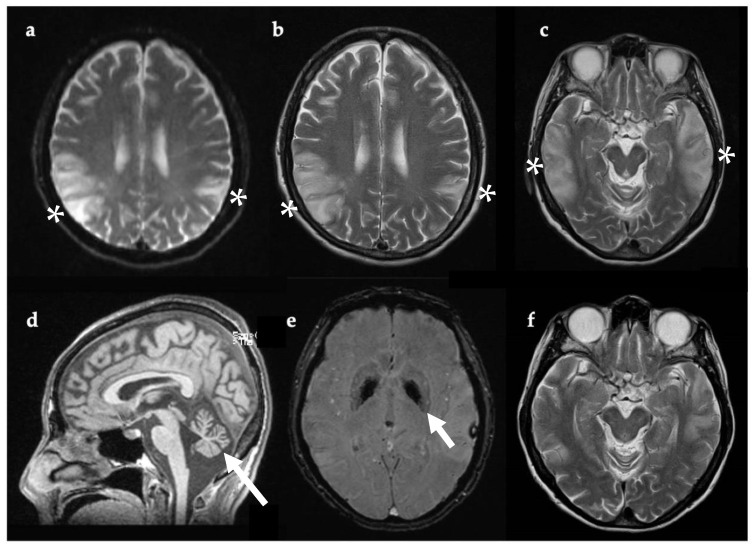
Axial (**a**–**c**,**e**,**f**) and sagittal (**d**) brain MRI, 11 days after symptom onset (**a**–**e**) and one year later (**f**). Bilateral temporoparietal cortical and gyral swelling shown with white asterix in (**a**), DWI (**b**), and (**c**) T2w. Evidence of cerebellar atrophy (**d**, white arrow). SWI sequence showing pallidal mineralization (**e**, white arrow). MRI one year later (**f**) showed regression of lesions previously evident (**c**). Abbreviations: DWI, diffusion-weighted imaging; SWI, susceptibility-weighted imaging; T2w, T2-weighted imaging.

Past medical history included type 1 diabetes diagnosed at 41 years, and Grave’s thyrotoxicosis. In the weeks preceding her hospital admission she had been suffering with headaches and fatigue. On the day she presented to the Emergency Department (ED), she had become agitated and developed visual hallucinations (pacing, undressing, checking the front door repeatedly, shouting out, and picking at thin air). No seizure activity was documented. She was acidotic (pH 7.25, pCO2 5.96 kPa, pO2 4.62 kPa, lactate 4.3 mmol/L, base excess—7.4 mmol/L, HCO3—17.7 mEq/L) and erythrocyte sedimentation rate (ESR) was elevated (43, reference range 0–30 mm/hr). She was not febrile. CK and blood lactate were increased (185 IU/L, normal range 26–140 IU/L, and 4.68 mmol/L, normal range 0.5–2.2 mmol/L, respectively). Viral encephalitis was suspected given the abrupt onset of headache, confusion, and increased ESR. Intravenous acyclovir was administered for four days prior to the results of lumbar puncture, which showed acellular CSF and negative viral PCR for HSV1/2, VZV, and enterovirus. Anti-NMDA encephalitis was also suspected given the neuropsychiatric symptoms. However, anti-NMDA receptor antibodies were negative in blood and CSF, as were other antibodies for autoimmune encephalitis, including CASPR2 and LGI1. EEG revealed periodic right temporal waves, while brain MRI confirmed large areas of non-arterial cortical/subcortical hyperintensities and swelling affecting the left temporal and biparietal regions, consistent with MELAS (Figure 2).

Muscle biopsy of left vastus lateralis showed changes compatible with PMD, including an excess of mitochondria in muscle fibres and COX-negative fibres. There were no ragged red fibres. Spectrophotometric analysis of the muscle homogenate, corrected for citrate synthase enzyme activity, confirmed decreased complex I (0.073, reference range 0.104–0.268) and complex IV (0.008, reference range 0.014–0.034) enzyme activities. Molecular analysis confirmed the m.3243A > G mutation in blood (6% heteroplasmy) and skeletal muscle tissue (74% heteroplasmy).

Following discharge, she returned to her premorbid baseline. Unfortunately, she has suffered two further SLEs over the past four years and has developed cognitive decline and gait ataxia.

### 3.3. Case 3

A 35-year-old man of white European heritage developed an acute episode of exertional rhabdomyolysis, which required transient renal replacement. He was subsequently diagnosed with hypertrophic cardiomyopathy and uncontrolled diabetes (haemoglobin A1c 14.7%, reference range 4–6% total haemoglobin). At that stage he was found to be a carrier of the m.3243A > G mutation (heteroplasmy 23% in blood).

At the age of 37 years, he presented to the ED following a tonic-clonic seizure. CT brain was normal and he was discharged.

Two weeks later, he re-presented to the ED with acute onset expressive aphasia. There was no associated weakness, visual problems, dysphagia, gait disturbance, or seizure activity, and he was normotensive. ECG was in sinus rhythm. Brain CT showed a new hypodensity involving both the grey and white matter within the left temporal lobe, consistent with a subacute left middle cerebral artery (MCA) infarct. However, CT angiogram did not identify a stenosis or occlusion of the proximal intracranial arteries. The onset of symptoms was unclear, so intravenous thrombolysis was not administered, and he commenced aspirin and clopidogrel in addition to atorvastatin. Echocardiogram confirmed hypertrophic cardiomyopathy. During his admission, he suffered several generalised tonic–clonic seizures and recurrent strokes also involving the occipital lobe, despite the antiplatelet therapy. Interictal EEG did not show epileptic activity, but there was an excess of slow activity over the left hemisphere, in keeping with focal cerebral dysfunction at this site.

**Figure 3:**
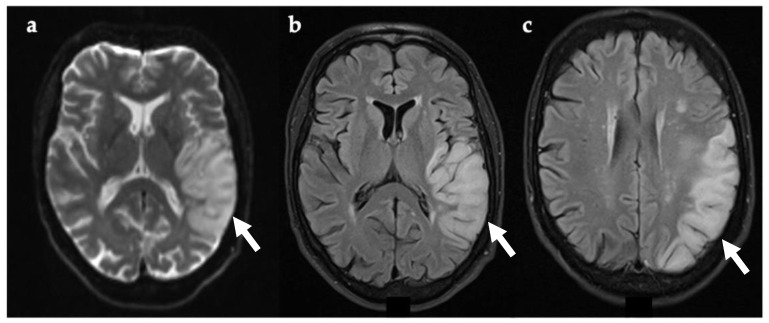
Axial brain MRI 24 h (**a**,**b**) and seven days (**c**) after symptom onset. Evidence of restricted diffusion in left temporal lobe (DWI) (**a**, white arrow). T2-FLAIR showing cortical and gyral swelling in left temporal lobe (**b**, white arrow). Image (**c**, white arrow) was taken seven days after symptom onset and shows extension of lesion on the left occipital lobe. Abbreviations: DWI, diffusion-weighted imaging; FLAIR, fluid attenuated inversion recovery imaging.

Brain MRI (Figure 3) undertaken 24 h after symptom onset confirmed an acute/subacute lesion in the left temporal lobe, which exhibited restricted diffusion on DWI. A follow up brain MRI performed after seven days showed extension of the lesion into the left occipital lobe (Figure 3), compatible with a mitochondrial stroke-like lesion. Levetiracetam was initiated and antiplatelets were discontinued. After starting antiepileptic treatment, seizures stopped and no further SLEs were observed. Since discharge, he has been well and his expressive aphasia has improved.

### 3.4. Case 4

A 44-year-old woman of white European background developed acute-onset slurred speech and intermittent twitching of the left cheek and hand. She attended the ED the following day after developing left-sided arm weakness and facial droop. On admission, she was apyrexial and alert, but speech was severely dysarthric. There was evidence of left-sided upper motor neuron facial weakness and reduced power in the left arm. Ongoing twitching of the left arm and face was resistant to anticonvulsants, progressed to focal refractory status, and she was transferred to the intensive therapy unit (ITU).

Past medical history included three similar presentations from the age of 35 years, which prompted a diagnosis of steroid-responsive CNS vasculitis based on clinical picture and brain imaging, and she was treated with steroids and cyclophosphamide. She experienced one focal seizure each month, treated with lamotrigine and clobazam. Brain biopsy undertaken at 35 years showed occipital lobe gliosis only. There was a positive family history documented in her mother with recurrent strokes, hearing problems, and similar radiological findings.

**Figure 4:**
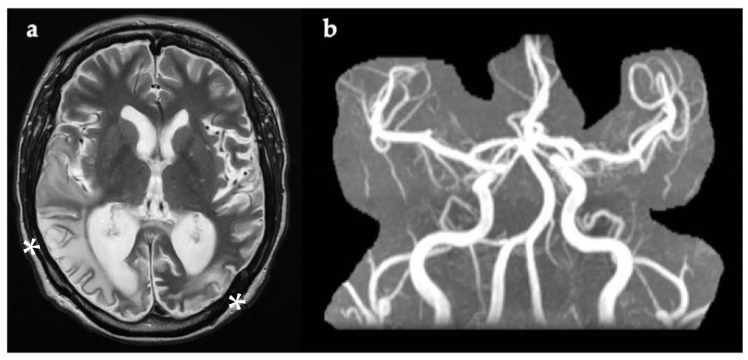
(**a**) Axial brain MRI image taken four days after symptom onset, showing multiple areas of white matter and cortical increased T2w signal within the posterior cerebral hemispheres (white asterix). DWI is not available. (**b**) Normal MRA with no evidence of narrowing of blood vessels, as usually present in CNS vasculitis. DWI, diffusion-weighted imaging; MRA, magnetic resonance angiography; T2w, T2-weighted imaging.

During the most recent admission, septic and vasculitis screens, including ESR and C-reactive protein, were normal. Brain MRI (Figure 4) showed multiple areas of increased T2w signal in the cortex and white matter of the posterior cerebral hemispheres, with overlap between old and recent lesions. Magnetic resonance angiography (MRA) was normal (Figure 4). CSF was acellular with normal protein and glucose levels, but raised lactate (4 mmol/L, reference range 1.1–2.4 mmol/L). CSF viral PCR was negative. Status epilepticus was treated with intravenous diazepam and clobazam, levetiracetam, and lamotrigine.

The personal and family history of recurrent strokes and epilepsy, MRI findings, and increased CSF lactate, led to clinical re-evaluation of the case and MELAS was considered. The m.3243A > G mutation was detected in blood, urinary epithelial cells, and brain (formalin-fixed, paraffin-embedded tissue) with a heteroplasmy level of 5%, 46%, and 63%, respectively. The patient returned to her pre-admission baseline and has been stable since discharge.

## 4. Discussion

The clinical and radiological features of SLEs are highlighted in Table 2.

Despite improved non-invasive diagnostic methods, such as next-generation sequencing (NGS), the diagnosis of PMDs is challenging, as demonstrated by the four cases presented. Clinical manifestations of MELAS are highly variable and some cardinal features, such as SLE, can mimic other acquired neurologic disorders, which may delay diagnosis and appropriate management.

The role of muscle biopsy in the diagnosis of MELAS has diminished with the routine application of diagnostic NGS. Sequencing the entire mitochondrial genome can be performed in leukocytes or urinary epithelial cells. A further important consideration in PMDs caused by mtDNA mutations is the physiological phenomenon of heteroplasmy (i.e., the coexistence of both mutated and wild-type mtDNA within the same cell). Heteroplasmy can vary across and within tissues, which may influence clinical phenotype and disease severity [17]. Heteroplasmy levels in leucocytes decrease with age [18]; thus, mutant mtDNA may be undetectable in blood taken from older subjects, as highlighted by Case 1. Further genetic analysis of a second tissue should therefore be undertaken if the clinical index of suspicion is high. The urinary epithelial cells can be used for this purpose to avoid more invasive procedures (e.g., muscle biopsy) and the heteroplasmy levels in urinary sediment have been shown to correlate more closely with post-mitotic tissues (e.g., skeletal muscle tissue) than leukocytes [19,20,21]. Indeed, in our cases the mutation load in urine was much higher than in blood, more accurately reflecting the severity of the phenotypes.

The m.3243A > G pathogenic variant in *MT-TL1* is detected in approximately 80% of subjects with MELAS [22]. Additional causes include other pathogenic variants in *MT-TL1* (e.g., m.3271T > C and m.3252A > G) and *MT-ND5* (m.13513G > A). Mutations in nuclear genes, including *POLG* [23,24], *FASTKD2* [25], and *MRM2* [26], are a less common but important cause of mitochondrial strokes. Consequently, a multigene panel or exome sequencing is recommended if a mitochondrial stroke is suspected once a pathogenic mtDNA variant in a post-mitotic tissue is excluded.

The first clinical criteria for the diagnosis of MELAS were published in 1992 [15] and included episodes of SLE prior to 40 years. However, there is increasing evidence that SLEs may occur at more advanced age [27,28,29], as seen in Cases 1 and 2. The onset of symptoms does not tend to be hyperacute, as with thromboembolic strokes; rather, the focal deficits usually evolve over days. The presence of seizure activity also supports a mitochondrial (rather than a vascular) stroke [16,30]. Seizures can also occur without overt focal neurological deficits, as exhibited by Case 1 who had an isolated cluster of seizures prior to an SLE. The frequency of seizures in MELAS patients is estimated at 71% to 96% [16]. Seizure subtypes are heterogeneous with no pathognomonic features. Focal seizures are more common than generalised and EEG abnormalities tend to arise from the occipital, frontal, and temporal lobes. The mean age of onset is variable, with an average of 23 years [31]. The presence of epilepsy in MELAS is associated with a poor prognosis, particularly when they occur early in the disease course, if they are associated with status epilepticus, or if they are refractory to anticonvulsants [31,32].

MELAS is a multisystemic disease. Consequently, when a mitochondrial stroke is suspected, other clinical manifestations of mitochondrial pathology should be excluded. These include diabetes, cardiomyopathy, and/or deafness. However, the absence of additional organ involvement should not deter further genetic investigations for MELAS, as SLE may be the first clinical manifestation [33]. Increased CSF lactate supports a diagnosis of MELAS, as exhibited in Case 1 and 4, although these results should be interpreted with caution, as CSF lactate may also increase following a seizure or an ischemic cerebral event [34,35].

The differential diagnosis between ischemic stroke and SLE can be challenging during the acute phase, even in patients with a pre-existing diagnosis of PMD (as in Case 3). Therefore, it is important to supply medical warning information to patients when attending local units with limited experience of rare diseases. This challenge in the diagnosis is partly due to overlap in clinical presentation, but also because of shared risk factors in both patient groups; specifically, diabetes and/or cardiomyopathy. Indeed, stroke mimics account for up to 25% of stroke admissions and brain MRI is the modality of choice to distinguish vascular from non-vascular events [36]. Stroke-like lesions caused by mitochondrial SLE do not necessarily localize within vascular territories and can evolve over weeks with migration to adjacent brain regions [37]. The lesions (and neurological deficits) can resolve (as occurred in Case 2), but they can also recur. Cortical and subcortical white matter are often implicated, usually with sparing of the deep white matter [37]. During the acute phase, lesions show restricted diffusion, without reduction in apparent diffusion coefficient (ADC), unlike acute ischemic strokes [38]. This suggests vasogenic (rather than cytotoxic) oedema [39], although the precise pathophysiological mechanism remains unclear. Two major theories have been proposed [40]: first, the vascular hypothesis, which suggests mitochondrial proliferation is responsible for an ischemic angiopathy in the smooth muscle layer of small arteries; and second, the mitochondrial cytopathy theory, which proposes that mitochondrial dysfunction triggers the energy failure of brain tissue with consequent neuronal damage. Ictal activity has also been linked with brain damage during SLEs via neuronal hyperexcitability [1].

The uncertainty surrounding underlying pathophysiology has challenged the development of universally accepted management guidelines, although progress has recently been made in this regard [1]. Aggressive treatment of seizures (as reported in Case 3, in which the seizure treatment considerably improved the outcome), adequate fluid management, and correction of lactic acidaemia are all recommended. Intestinal pseudo-obstruction, as reported in Case 1, is frequently reported in SLE and can be associated with gastroparesis. It is therefore important to continually evaluate bowel function and actively manage dysmotility conservatively, via insertion of a wide-bore nasogastric tube. Randomized, placebo-controlled, double-blind clinical trials of L-arginine have not been undertaken. Consequently, routine administration of L-arginine is dependent on local clinical guidelines. With the exception of valproic acid (which is contraindicated as it inhibits mitochondrial bioenergetics) [41], management of seizures follows standard treatment guidelines.

SLEs evolve over days and are often associated with headache, behavioural changes, and visual hallucinations. Consequently, viral, immune-mediated encephalitis and cerebral vasculitis should be considered [42,43,44]. However, an acellular spinal tap with negative viral PCR, indicative MRI features, including migration of brain lesions and a lactate peak at MR spectroscopy, a positive personal or family history of similar repeated relapsing/remitting neurological episodes, seizures, diabetes, and/or deafness, help distinguish mitochondrial strokes from other inflammatory and immune-mediated CNS disorders. Furthermore, behavioural and psychiatric manifestations in SLEs may be prominent, as shown in Case 2. A diagnosis of MELAS should therefore be taken into consideration in subjects with an acute/subacute onset of psychiatric disease in the presence of other red flag features.

Finally, the diagnosis of MELAS has important implications for the proband, who will benefit from screening tests and reproductive options, given offspring will be obligate carriers (if the proband is a female) [45], but also for extended family members, who may opt for cascade diagnostic testing.

## 5. Conclusions

In conclusion, we highlight the challenges faced when diagnosing mitochondrial strokes through the clinical presentations of four individuals with SLEs caused by MELAS. PMDs should be considered in anyone with acute/subacute onset of focal neurological symptoms, with or without seizures, presenting at any age. Typical MRI findings, the coexistence of multisystemic features, including diabetes, deafness, and/or cardiomyopathy, or a positive maternal family history, support the diagnosis of MELAS, but are not always present. Establishing a diagnosis has important implications for management during the acute stages of SLE, including avoiding unnecessary and potentially harmful treatments, while identification of the underlying molecular defect enables genetic counselling, cascade testing, and discussion concerning reproductive options.

## Figures and Tables

**Table 1:** Clinical, radiological, and molecular characteristics of four subjects with stroke-like episodes caused by the m.3243A > G mutation in *MT-TL1*.

Case	Het ^1^	Age of 1st SLE (Latency to PMD Diagnosis)	Neurological Presentation of SLE	Brain MRI		EEG		Other PMD Symptoms	Differential Diagnosis
				**Days ^2^**	**Findings**	**Days ^2^**	**Findings**		
**1**	B: neg U: 29%	51 y (10 y)	Imbalance, drowsiness, seizures	21	Lesions in temporo-parieto-occipital lobes bilaterally	15	Occasional left temporal sharp waves	Epilepsy, diabetes, deafness	Viral encephalitis
**2**	B: 6% M: 74%	51 y (6 m)	Headache, confusion, visual hallucination, behavioural changes	11	Bilateral temporo-parietal cortical lesions, pallidal mineralisation and cerebellar atrophy	7	Periodic right temporal waves	Diabetes	Autoimmune/ Viral encephalitis
**3**	B: 23%	37 y (PMD diagnosed before)	Expressive aphasia, seizures	1	Left temporal lobe lesion	10	Slow activity on the left hemisphere	Hypertrophic CM, diabetes	Left MCA infarct
**4**	B: 5% U: 46% Br: 63%	35 y (9 y)	Left-sided UMN VIIth cranial nerve palsy. LUL weakness, seizures	4	Multiple areas of white matter and cortical increased T2w signal within the posterior cerebral hemispheres	3	Spike and waves and sharp waves over the right frontal areas	None	CNS vasculitis

^1^ Heteroplasmy level of m.3243A > G, *MT-TL1*. ^2^ Days from symptom onset to brain MRI or EEG. Abbreviations: B, blood; Br, brain; CM: cardiomyopathy; LUL, left upper limb; M, muscle; m, months; MCA, middle cerebral artery; neg, negative; PMD, primary mitochondrial disease; SLE, stroke-like episode; U, urinary epithelial cells; UMN, upper motor neuron; y, years.

**Table 2:** Clinical and radiological characteristics of stroke-like episodes due to MELAS.

Clinical Features	Radiological Features
Onset of symptoms is generally subacute (days) and clinical symptoms reflect lesion site.	SLE lesions inconsistent with vascular territories.
Age of onset is variable, but usually before the age of 40 years [15].	Lesions usually in posterior brain regions (parietal/occipital lobes).
Epileptic seizures occur in 71% to 96% of MELAS [16].	DWI hyperintensity in acute phase (as in ischemic lesions).
Common symptoms are headache, cortical blindness, hemianopsia, hemiplegia, and behavioural changes.	Involvement of cerebral cortex, with swelling on FLAIR.
Neurological symptoms are often reversible.	Lesions can migrate/extend over weeks.
Coexisting diabetes, hearing loss, short stature.	Radiological lesions are often reversible.
Positive maternal family history, especially of diabetes and/or deafness.	No reduction in ADC (unlike ischemic lesions).
The mutation (e.g., m.3243A > G, *MT-TL1*) may not be detectable in blood in older patients and where possible a second tissue, e.g., urinary epithelial cells or skeletal muscle, should be tested. If negative, whole mtDNA sequencing, multigene panel, and/or exome sequencing are recommended.	Elevated lactate peak at 1.3ppm and decreased NAA on MRS.

Abbreviations: ADC, apparent diffusion coefficient; FLAIR, fluid-attenuated inversion recovery; DWI, diffusion-weighted images; MRS, magnetic resonance spectroscopy; NAA, N-acetylaspartate; SLE, stroke-like episode.

## Data Availability

The data presented in this study are available on request from the corresponding author.
